# Angioinvasive mucormycosis and paradoxical stroke: a case report

**DOI:** 10.1099/jmmcr.0.005048

**Published:** 2016-08-30

**Authors:** Roxana Mititelu, Samuel Bourassa-Blanchette, Karan Sharma, Virginia Roth

**Affiliations:** ^1^​ McGill University, Montreal, Quebec, Canada; ^2^​ Department of Internal medicine, The Ottawa Hospital, Ottawa, Ontario, Canada; ^3^​ Department of Infectious Diseases, The Ottawa Hospital, Ottawa, Ontario, Canada

**Keywords:** aphasia, Mucormycosis, infectious endocarditis, stroke, PFO, antifungal, iron chelator

## Abstract

**Introduction:**

*Rhizopus* typically results in acute, aggressive and angioinvasive infection, particularly in immunosuppressed individuals. Risk factors include immunosuppression in haematologic malignancy, uncontrolled hyperglycemia, iron overload states, and older chelator agents such as deferoxamine.

**Case Presentation:**

We describe a case of a 33-year-old female with transfusion-dependent beta thalassemia who was started on intravenous deferiprone therapy and subsequently presented with a retropharyngeal abscess. Despite intravenous broad spectrum antibiotics, she continued to deteriorate and developed aphasia. A CT scan of her head showed multiple hypodensities. Blood cultures grew *Rhizopus *species and a subsequent transesophageal echocardiogram showed a mass in the right atrium with a patent foramen ovale.

**Conclusion:**

Although deferiprone, a newer iron chelator agent, has antifungal properties *in vivo*, this case illustrates that angioinvasive *Rhizopus *infections can occur in patients treated with deferiprone.

## Introduction

Mucorales are fungi that are ubiquitous in nature, commonly found on decaying plants and in soil. The genera commonly causing human infection include *Rhizopus*,* Mucor *and *Rhizomucor*. Overall, mucormycosis rarely cause infection in humans, and most patients with invasive disease have some underlying disease that predisposes to the infection. The most common predisposing illnesses are underlying immunosuppression in the form of haematological malignancies, solid organ transplantation, haematopoietic stem cell transplantation and diabetes mellitus with ketoacidosis. A less common risk factor for invasive mucormycosis is iron overload states and treatment with iron chelators. The deferoxamine iron chelator acts as a siderophore for *Mucor *species increasing iron uptake which in turn enhances fungal growth and invasiveness. This is in contrast to newer iron chelators such as deferasirox and deferiprone which do not act as siderophores for the fungi and therefore are thought to decrease the risk of mucormycosis. The DEFEAT mucor study has been the biggest study to date looking at the benefit of combination liposomal amphotericin B (LAmB) and deferasirox in patients with proven or probable mucormycosis. However, this study showed that death in the group receiving the newer iron chelating agent, deferasirox, was higher when compared to placebo (Spellberg *et al.*, 2012). Here, we present an interesting case of a 33-year-old female with iron overload on deferoxamine therapy with invasive mucormycosis.

## Case report

A 33-year-old female with transfusion-dependent beta thalassemia major resulting in severe iron overload, amenorrhea, extramedullary haematopoiesis, and osteoporosis with rib fractures, presented to our tertiary care hospital with febrile neutropenia. Two years prior, she began IV deferoxamine therapy at the maximal dose of 50 mg kg^−1^ day for severe iron overload. However, three months prior to admission, she was switched from IV deferoxamine to deferiprone due to her cardiac MRI showing persistently high cardiac iron levels despite 18 months of deferoxamine therapy.

On admission, she presented with a two-day history of sore throat and fever. She had no known infectious contacts. She travelled to India for her work six months prior to presentation, but remained well during and after the trip. A computed tomography (CT) scan of her neck revealed a retropharyngeal abscess, which was treated empirically with IV piperacillin-tazobactam and dexamethasone. She improved clinically over the next 48–72 h with resolution of her fever and improvement in her neck pain. An initial chest X-ray was normal and blood cultures were negative.

Seven days after admission, she experienced recurrent fever with pleuritic chest pain and shortness of breath. A chest X-ray revealed a right upper lobe and left lower lobe consolidation and her antimicrobial therapy was broadened to azithromycin, meropenem and vancomycin. The tip of her peripherally inserted central catheter (PICC) line was culture positive for coagulase-negative *Staphylococcus*, but blood cultures remained negative. An ultrasound of her right arm initially revealed no deep vein thrombosis (DVT). However, four days later, her right arm was erythematous and swollen, and a repeat ultrasound revealed a right innominate vein DVT. Enoxaparin was started.

**Fig. 1.**
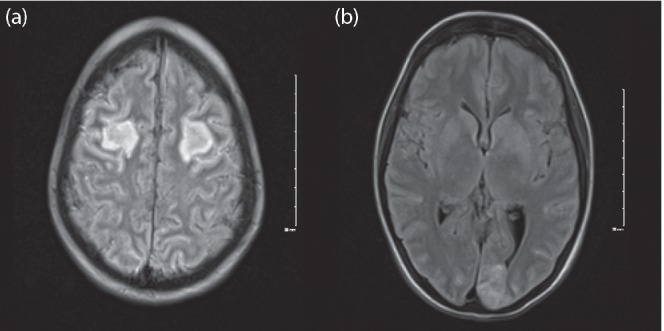
(**a**) MRI axial T2 FLAIR image demonstrating bilateral frontal acute infarction (b) MRI axial demonstrating acute left occipital and subactue caudate stroke.

Two weeks after admission, the patient developed word-finding difficulties and a CT head scan showed ill-defined hypodensities in the frontal lobe bilaterally and the left occipital lobe believed to be secondary to ischemic versus infectious aetiology. She developed focal seizures with dyscognitive features which manifested as the patient suddenly became unable to look to her left with right-sided facial twitching and a decreased level of consciousness. Neurology was consulted and the patient began therapy with levetiracetam with good response. An MRI was performed a few days after which demonstrated bilateral frontal and left occipital cortical/subcortical acute infarction as seen on CT the day of her ictal episode, as well as new small areas infarct involving the left caudate and right parietal cortex (Fig. 1a). Repeat blood and bronchoalveolar lavage (BAL) cultures were negative for bacterial and fungal organisms. However, given her persistent febrile neutropenia and possible brain abscesses, voriconazole was added to her empiric antimicrobial regimen.

**Fig. 2.**
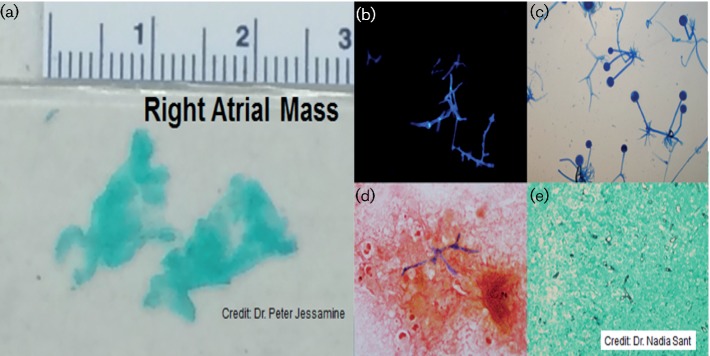
Gross section of the intracardiac mass (a); cotton blue mount showing characteristic microscopic morphology of *Rhizopus* isolate (b); calcofluor white mount showing hyphae in the tissue (c); non-septate hyphal elements in the resected mass stained with Gram stain (d); Gomori methenamine silver stain showing aseptate hyphae in the tissue (e).

Meanwhile, the clinical status of the patient continued to decline and the critical care team was consulted for sinus tachycardia, tachypnea, leukocytosis (white blood cell count of 63.3×10^9^ l^−1^), and acute kidney failure, including hyperkalemia of 7.2 mmol l^−1^ and a creatinine of 144 mmol‌ l^−1^. She was transferred to the ICU and underwent urgent dialysis for the severe hyperkalemia. A repeat CT head scan was ordered and showed no evidence of haemorrhage and the same previously seen hypodensities. A lumbar puncture was unremarkable. An MRI of her brain showed the same bilateral frontal and left occipital cortical and subcortical acute infarctions with small areas of infarct of the left caudate and right parietal cortex with no mycotic aneurysm and no brain abscess. While a transthoracic echocardiogram (TTE) was unremarkable except for an incidental finding of an atrial septal aneurysm, a transesophageal echocardiogram (TEE) revealed the presence of an intracardiac mass of the right atrium infiltrating a patent foramen ovale (PFO) and measuring 4.1×1.2 cm in the right atrium (Fig. 2a). There was no valvular pathology. Given these findings, along with the filamentous fungus found on blood culture, the anti-fungal treatment was broadened to amphotericin B. The patient was then transferred to another tertiary care hospital for cardiac embolectomy. In the operating room, a large amount of infected tissue was noted to be sitting at the level of the atrial septum in the right atrium. Microscopic mount of the resected tissue (Fig. 2b) under histopathological examination showed hyphae with calcofluor white mount (Fig. 2c), and non-septate hyphal elements with Gram stain (Fig. 2d). While, Gomori methenamine silver stain showed aseptate hyphae in the tissue (Fig. 2e). These findings are consistent with *Rhizopus* species. The interatrial septum was noted to be largely intact with likely only a small PFO. When the fossa ovalis was incised, a pocket of purulent material was entered, which was believed to be an abscess-like cavity. This cavity was then debrided. The fossa ovalis was completely resected and patched with bovine pericardium.

Twelve days post-operatively, the patient developed new onset left-sided weakness. Repeat imaging with CT angiogram identified the presence of a small (2.2×1.4 mm) mycotic aneurysm of the right lenticulostriate artery with an associated 3 cm intracerebral haemorrhage (Fig. 1b). She then underwent a right pterional craniotomy and mycotic aneurysm clipping without any complications. Incidentally, an MRI showed the previously described lesions within the frontal lobes and left occipital lobe which demonstrated signal MR characteristics compatible with fungal abscesses secondary to septic emboli. There was also new infarction that had developed within the right caudate nucleus, lentiform nucleus and internal capsule, as well as in a small region lateral to the right frontal horn. Finally, there was subarachnoid and subdural haemorrhage that remained comparable in size and extent to on the recently performed CT head. There was no significant hydrocephalus and no herniation. Imaging also showed progression of a necrotizing pneumonia with adjacent rib osteomyelitis (Fig. 1c, d). Bronchoscopy revealed that she bled into the involved lung segment requiring embolization of five arteries under interventional radiology. Caspofungin was added to the amphotericin B. Yet despite the worsening radiological findings, the neurological status of the patient gradually improved and she recovered some function on her left side.

The patient recovered slowly from her acute hospitalization, requiring an extensive stay in the rehabilitation unit after having been severely weakened by her illness. She returned home more than four months after her admission. Caspofungin and amphotericin B were continued in combination for a total of five months. Caspofungin was discontinued after repeat imaging showed satisfactory response to treatment. Amphotericin B was discontinued after a total of six months of treatment, with no further step-down or oral antifungal therapy.

## Discussion

Deferoxamine is one of the first iron chelating agents developed initially from the siderophore of *Streptomyces pilosus* (Brittenham *et al.*, 1994). Siderophores are strong iron chelating agents that are utilized by different micro-organisms such as plants, fungi and bacteria to take up iron as a growth factor. Current indications for their use include iron overload states such as haemochromatosis and transfusion-dependent thalassemia. Prior research has shown that both mucormycosis as well as leukopenia are dose-dependent side effects (Van Cutsem & Boelaert, 1989; Kontoghiorghes, 1995). Our case also demonstrates this, as our patient was on the maximal dose of the drug prior to presenting with both neutropenia and an invasive fungal infection. The mechanism behind the increased risk of infection for mucorales in patients treated with deferoxamine especially *Rhizopus orizae* is thought to be caused by the ability of the fungal species to use the deferoxamine as a xenosiderophore to increase its own iron uptake resulting in growth and proliferation (Boelaert *et al.*, 1993). Interestingly, there have been case reports where treatment for mucormycosis not only includes amphotericin B, but can also include deferasirox, a newer, orally available, iron-chelating agent that cannot be utilized by mucorales as a xenosiderophore (Kontoghiorghes, 1995). The leukopenia and the clinically important neutropenia associated with the intravenous iron chelation therapy are believed to be related to an arrest in cell maturation of the progenitor cells of granulocytic lineage (Piga *et al.*, 2005). This effect is believed to be both dose- and time-dependent. The leukopenia effect itself could also be playing a role in the mucorales infection, which would otherwise be extremely rare to present in immunocompetent patients. Interestingly in this case, the patient did not become neutropenic while on deferoxamine, but only one month after initiation of deferiprone was started.

Newer iron chelators such as deferasirox and deferiprone do not act as siderophores for the fungi and therefore are thought to the decrease the risk of mucormycosis. Furthermore, use of newer chelators like deferasirox in murine models has actually shown a benefit when used to treat mucormycosis. The DEFEAT mucor study included twenty patients randomized to receive liposomal amphotericin B (LAmB) plus deferasirox or LAmB plus placebo. Interestingly, death at 90 days was higher in patients who received deferasirox when compared to placebo (82 vs 22 %, respectively) (Spellberg *et al.*, 2012). However, patients in the deferasirox group had more associated comorbid conditions possibly resulting in poorer outcomes.

Mucormycosis is characterized by its ability to invade blood vessels, and in this case it was angioinvasive in the brain, lungs, bones and heart of the patient. Specifically, cardiac mucormycosis is characterized by fungal thrombi containing invasive nonseptate branching hyphae that can penetrate the myocardium and endocardium with subsequent infarction of the tissue. The infarction is often due to the fungal thrombi occurring inside the small myocardial arteries and veins (Jackman & Simonsen, 1992). A risk factor for acquiring endovascular mucormycosis is IV drug use through spores on contaminated needles (Roden *et al.*, 2005). Cardiac mucormycosis can result in pericarditis, myocarditis and endocarditis (both native and prosthetic valves). Native valve endocarditis has been reported to involve the aortic, mitral and pulmonic valve and has been described in cardiac surgery patients (Jackman & Simonsen, 1992; Virmani *et al.*, 1982). Septic emboli have been reported in prosthetic valve mucormycosis endocarditis (Virmani *et al.*, 1982). Interestingly, deferoxamine therapy has been demonstrated as the risk factor that predisposes to the highest level of generalized dissemination infection (23 %), and native valve endocarditis has only been reported in disseminated mucormycosis, similar to our patient (Roden *et al.*, 2005; Jackman & Simonsen, 1992). The treatment for mucormycosis involves removing infected tissue if possible and timely antifungal therapy. In a study examining zygomycosis, surgical resection of the infected tissue showed increased survival rates of up to 70 % when combined with antifungal therapy (Roden *et al.*, 2005).

Our patient posed a challenging treatment dilemma with unique parameters to consider, once she developed the right atrial thrombus in the setting of a PFO. Similar to our case, there are multiple case reports of impending paradoxical emboli in the literature (Meacham III *et al.*, 1998; Falk *et al.*, 1997; Caes *et al.*, 1995). The diagnosis of a stroke secondary to a paradoxical embolism via PFO or an atrial septal defect (ASD) is a diagnosis of exclusion and thus should be confirmed with an echocardiogram with agitated saline at rest, while coughing, and with the Valsalva maneuver. In cases where there is a risk for paradoxical embolism, embolectomy is preferred when it can be performed by an experienced surgeon working in a facility with cardiopulmonary bypass capacities (Myers *et al.*, 2010). Embolectomy does have a theoretical advantage since the surgical approach allows for the opportunity to repair the PFO, thereby reducing the risk of paradoxical embolism and subsequent stroke.

In summary, this case demonstrates the complex multidisciplinary approach required for patients who develop invasive mucormycosis infection secondary to deferiprone treatment. This unique case demonstrates the occurrence of fungal native endocarditis in the setting of disseminated mucormycosis and further complicated by a patent foramen ovale. Despite the high mortality associated with mucormycosis, our patient recovered close to baseline status with the coordinated care of a diverse set of specialties including haematology, ENT, infectious disease, critical care, cardiac surgery and neurosurgery. Appropriate management for complex patients such as this involves appropriate antifungal treatment and surgical removal of the vegetations.
